# A simple algorithm to assess patient suitability for Calypso‐seed implantation for four‐dimensional prostate localization

**DOI:** 10.1120/jacmp.v11i1.3107

**Published:** 2009-12-03

**Authors:** Randall J. Kimple, Eric M. Wallen, Raj Pruthi, Lawrence B. Marks

**Affiliations:** ^1^ Departments of Radiation Oncology University of North Carolina at Chapel Hill Chapel Hill NC 27599 USA; ^2^ Urologic Surgery University of North Carolina at Chapel Hill Chapel Hill NC 27599 USA; ^3^ Lineberger Comprehensive Cancer Center University of North Carolina at Chapel Hill Chapel Hill NC 27599 USA

**Keywords:** prostatic neoplasms, radiotherapy

## Abstract

To retrospectively determine the proportion of prostate cancer patients who are appropriate candidates for prostate localization with Calypso (Calypso Medical, Seattle, WA); to assess the accuracy of surface anatomy in predicting prostate depth; and to describe a simple clinical algorithm predicting patient's appropriateness for Calypso localization. Medical records and archived CT scans of all patients treated for localized prostate cancer at our institution between 2006 and 2007 were reviewed. Association between the feasibility of Calypso use, the depth of the prostate from the anterior torso, and a variety of anatomic factors were assessed (ANOVA, linear regression, and ROC). Patients were appropriate for the Calypso system in 91% of cases (localize and track, 52%; localize only, 39%). Strong correlation between greater trochanter location and the posterior prostate was seen ${(r}^{2}\;=\;0.91$, mean difference 0.6 cm). The negative predictive value of the greater trochanter measurements was 31%. Thirty‐one out of forty‐five patients (69%) who were deemed inappropriate for Calypso based on greater trochanter to anterior torso measurements were eligible on the basis of CT‐based measurements of prostate depth. Weight, BMI, waist circumference, and hip circumference correlated with distance from the prostate to the anterior torso and were predictive of Calypso appropriateness. All patients with weight ≤ 100kg,BMI≤ 30, or waist/hip circumference ≤ 100cm, were eligible for Calypso. Most prostate cancer patients are candidates for Calypso localization ± tracking. The greater trochanter to anterior torso distance underestimates the number of eligible patients. Weight, BMI and waist/hip circumference are good predictors for Calypso appropriateness.

PACS number: 87.63.‐d

## I. INTRODUCTION

**Figure 1:**
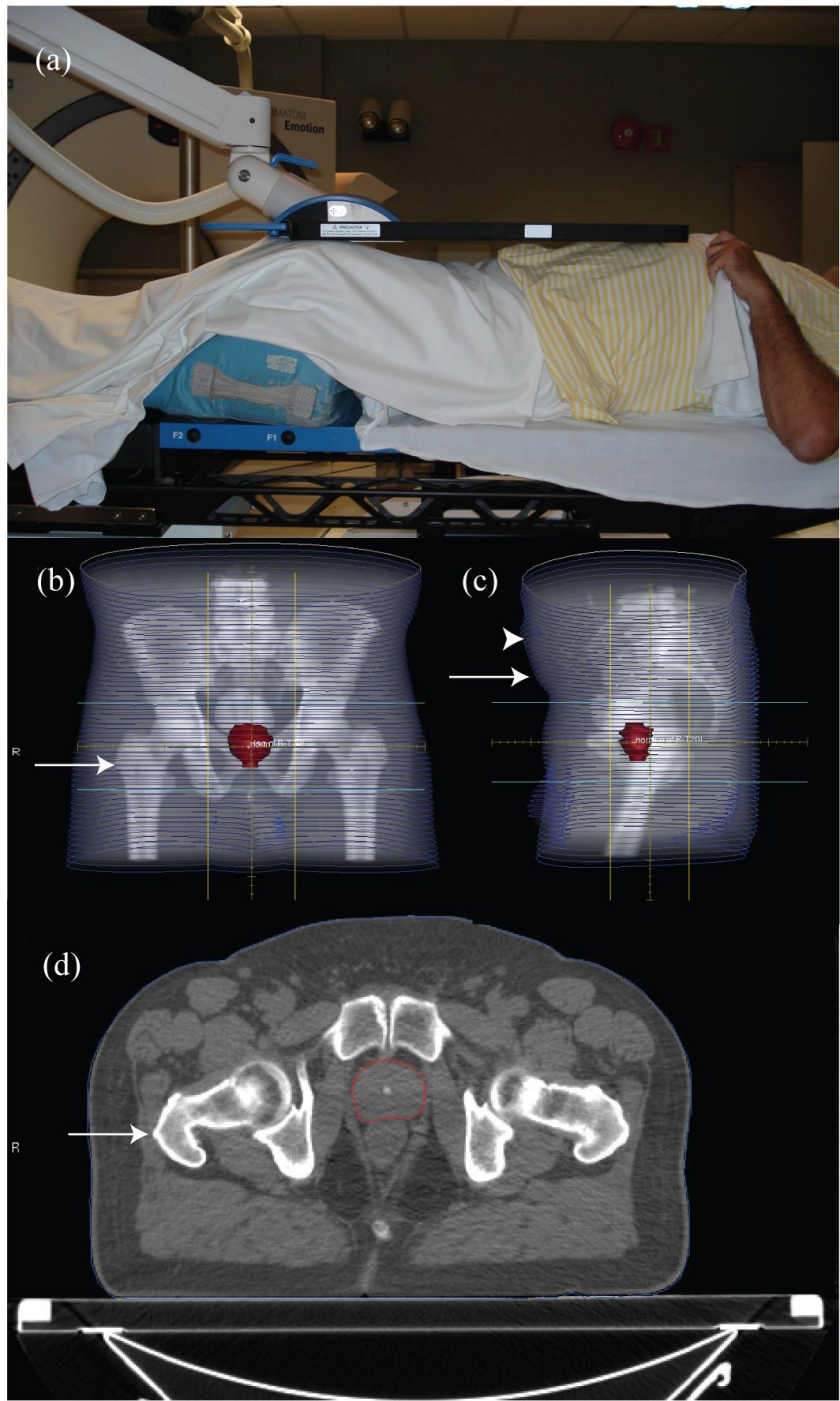
Setup of a patient with the Calypso array in place above the torso (a). AP (b) and lateral (c) digitally reconstructed radiograph from the CT simulation of a patient. Hip circumference was taken at the level of the greater trochanter (arrow in (b)). Waist circumference was taken at the level of the anterior superior iliac spine (arrow in (c)). The largest distance from the table to the anterior torso was also recorded (arrowhead in (c)). The distance from the table top to the greater trochanter (arrow in (d), anterior prostate, and posterior prostate were measured on an axial image.

Accurate localization of target tissues is a cornerstone of modern conformal radiation therapy (RT). The Calypso system (Calypso Medical, Seattle, WA) allows for localization and real‐time tracking of the prostate to improve the accuracy of RT delivery. Electromagnetic markers (i.e., Beacon transponders) implanted within the prostate, usually by the urologist (via a transrectal or transperineal approach with few reported complications^(^ ^1^ ^,^ ^2^ ^)^), are powered by a non‐ionizing oscillating electromagnetic field. The field is generated by an electromagnetic array that is placed anterior to the supine patient (Fig. 1(a)).^(^ ^1^ ^,^ ^3^ ^,^ ^4^ ^)^ The location of the “activated” transponders – and, hence, the prostate – is determined by triangulating their position relative to the array. An important limitation of the Calypso system is the presence of conductive interference from the tissue between the external array and the implanted transponders.

The accuracy of the system is inversely related to the depth of transponders within tissue^(3)^ so that intraprostatic placement of transponders is not appropriate in some large‐girth patients. It is important that physicians rapidly and accurately assess whether a patient is an acceptable candidate for the Calypso system to ensure appropriate implantation of the transponders as part of a multidisciplinary treatment approach.

While recent reports describe the use of the Calypso system in the setting of both single and multi‐institutional clinical trials,^(^ ^1^ ^,^ ^2^ ^,^ ^5^ ^)^ its applicability to routine clinical practice has not been studied. The manufacturer provides the users with guidelines to help determine which patients are most likely to be appropriate candidates for this technology based on the depth of the prostate relative to the anterior torso. The anterior torso is taken as a surrogate for the location of the array. While the manufacturer provides a worksheet to estimate the prostate depth based only on surface anatomy measurements, the applicability of this worksheet to routine clinical practice remain unclear. The aims of this study are to determine:

1. the fraction of prostate cancer patients predicted to be appropriate candidates for Calypso based on the manufacturer's worksheet and medical contraindications,
2. the accuracy of the surface anatomy‐based worksheets provided by Calypso in predicting the depth of the prostate, and
3. if a better predictive algorithm for prostate depth can be created based on simple clinical factors such as height, weight, and abdominal girth.

A simple algorithm that can be readily applied in the clinic may facilitate the rapid determination of Calypso appropriateness and obviate the need for pelvic‐imaging studies to make this determination.

## II. MATERIALS AND METHODS

In accordance with the principles and practices of our Institutional Review Board and in recognition of and compliance with HIPAA guidelines (United States Health Insurance Portability and Accountability Act of 1996), a retrospective chart review was performed to identify all patients treated with RT for intact localized prostate cancer at our institution in 2006‐2007. Only patients with archived treatment planning images were included.

Height, weight, and age at the time of the initial evaluation were taken from the medical record and used to calculate the body mass index (BMI). The archived treatment planning computed tomography (CT) scan was used to determine the waist and hip circumference at the level of the right greater trochanter and right anterior superior iliac spine, respectively (Figs. 1(b), 1(c)). The greatest anterior to posterior separation as determined by cross‐sectional imaging was recorded to estimate the position of the Calypso system localization array (Fig. 1(c)). The distance from the table top to the right greater trochanter, midline anterior prostate, and midline posterior prostate (Fig. 1(d)) were measured on CT images to minimize perspective distortion. Distances from the anterior‐most aspect of the torso to the greater trochanter, anterior prostate, and posterior prostate were calculated.

According to the manufacturer's recommendations, the transponders must be located no more than 21 cm below the array to “localize and track”, and no more than 27 cm below the array to use the “localize only” capabilities of the system. Calypso Medical provides a worksheet based on the distance from the greater trochanter to the anterior‐most aspect of the torso to estimate whether a patient is appropriate for use of their system. We used this worksheet to classify patients as 1) appropriate for localization and tracking (< 17 cm), 2) appropriate for localization only (17 cm ≤ X ≤ 23 cm), or 3) inappropriate for Calypso use (> 23 cm) on the basis of the greater trochanter to anterior torso distance. The 4 cm difference between the greater trochanter to anterior torso measurement and the array to transponder measurement is to allow for a slight air gap between the patient's skin and the array, and to account for respiratory movement of the abdomen and chest wall.

### A. Data analysis

To assess the fraction of patients who are predicted to be appropriate for Calypso based on the manufacturer's recommendations, exact 95% confidence intervals were calculated using GraphPad QuickCalcs (www.graphpad.com/quickcalcs/). To assess the accuracy of the surface anatomy‐based worksheets provided by Calypso in predicting the depth of the prostate, the estimated depth of the posterior prostate (based on the distance from the greater trochanter to the anterior torso as suggested by Calypso Medical^(6)^) was compared to the actual measured depth (from the CT images) by linear regression using GraphPad Prism version 5.01 (GraphPad Software, San Diego, CA). To assess if a better predictive algorithm for prostate depth can be created based on simple clinical factors, the association between the measured depth of the posterior prostate (from CT) and a variety of clinical factors were assessed using receiver operating curves and linear regression. The ability of these clinical measures to accurately identify patients as appropriate candidates for Calypso use (i.e. prostate depth within manufacturer recommendations) was assessed by calculating the sensitivity and specificity for selected cutpoints using GraphPad Prism. The accuracy of the manufacturer‐recommended approach (measurements from the greater trochanter) and these alternate clinical measures in their ability to accurately predict Calypso appropriateness were compared. To provide a true estimate of the proportion of patients for whom Calypso was appropriate, even patients ineligible for Calypso on the basis of anticoagulant use or hip prosthesis were included in all analyses.

## III. RESULTS

Ninety‐four patients who met the inclusion criteria were identified. Their mean age was 65.8 (range 45‐88). Mean weight (92.8 kg; range 55.1‐180.1 kg) and height (1.77 m; range 1.52‐1.91 m) were used to calculate BMI (average $29.7\;\text{kg}/m^{2}$, range $19.6‐51.0\;\text{kg}/m^{2}$). Five patients did not have their height recorded in the medical record and were excluded from the calculation and analysis of BMI. The remaining 89 patients were included in all analyses. The average cranial extent of the scans as measured from the center of the prostate was 20.8 cm (range 13.8–40.8) and 22% of scans extended ≥ 24cm cranial from the prostate. No differences were seen in measured variables between patients with a CT scan extending ≥ 24cm cranial to the prostate compared to those whose scan extended < 24cm (data not shown).

The median distance between the greater trochanter and the anterior torso was 16.3 cm (range 9.5‐28.5). Based on these measurements, patients were classified into one of three groups according to the manufacturer's preimplantation worksheet: 1) localize and track, 49/94 (52%, CI: 42%–62%); 2) localize only, 37/94 (39%, CI: 30%–49%); and 3) ineligible for implant, 8/94 (8.5%, CI: 4.2%–16%). In all, 17/94 (18.1%, CI: 11.5%–27.2%) of patients were ineligible for Calypso use (Table 1 on the basis of body habitus, prior hip replacement surgery (n=3), or anticoagulant use (n=6). One patient was ineligible on the basis of both body habitus and anticoagulant use.

**Table 1:** Reasons for patient ineligibility for Calypso Beacon transponder implantation.

*Reason for Ineligibility*	*N (%)*
Anticoagulant Use	6 (6.4%)
Hip Prothesis	3 (3.2%)
Body Habitus	7 (7.4%)
Anticoagulant Use & Body Habitus	1 (1.1%)
TOTAL	17 (18.1%)

**Figure 2:**
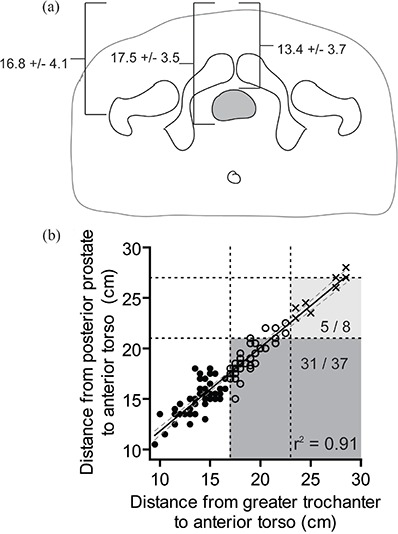
Mean (±standard deviation) distance from the greater trochanter, anterior prostate, and posterior prostate to the most anterior aspect of the torso (a); scatter plot of greater trochanter to anterior torso distance versus posterior prostate to anterior torso distance ${(r}^{2}\;=\;0.91)$ (b). The slope of the line is 0.83 (95% CI 0.77 to 0.88). Based on manufacturer‐recommended surface measurements: those appropriate for localize and track (solid circles), localize only (open circles), or ineligible for Calypso (crosses) are indicated. The recommended maximum distances between the greater trochanter and anterior torso to be appropriate for tracking (17 cm) or localization (23 cm) are indicated by vertical dashes. The maximum recommended distance between implanted transponder and the array to be appropriate for tracking (21 cm) and localization (27 cm) are indicated by horizontal dashes. Patients who would be misclassified as not appropriate for “localize and track” (dark shaded region) or ineligible for Calypso use (light shaded region) are indicated.

The greater trochanter lay between the anterior and posterior prostate in 72/94 (77%, CI: 67%–84%) cases. The average distance from the greater trochanter, posterior prostate, and anterior prostate to the anterior torso was 16.8 cm, 17.5 cm, and 13.4 cm, respectively (Fig. 2(a)). The distance from the greater trochanter or posterior prostate to the anterior torso was highly correlated (Fig. 2(b), $r^{2}\;=\;0.91,\;p\;<\;0.0001)$. Similarly, the distance from the greater trochanter or anterior prostate to the anterior torso was highly correlated $(r^{2}\;=\;0.91,\;p\;<\;0.0001$, data not shown).

The use of surface anatomy surrogates (greater trochanter to anterior torso) underestimates the proportion of patients who are eligible for localize and track. Thirty‐one of forty‐five (69%, CI: 54%–81%) patients who were ineligible for localize and track on the basis of greater trochanter measurements would have been eligible using a cutoff of 21 cm between the anterior torso and posterior prostate (dark shaded region in Fig. 2 (b)). An additional five of eight patients (62.5%, CI: 30%–86%) who were considered ineligible for Calypso would in fact have been appropriate candidates to use the Calypso system for localization only (light shaded region in Fig. 2(b)).

**Figure 3:**
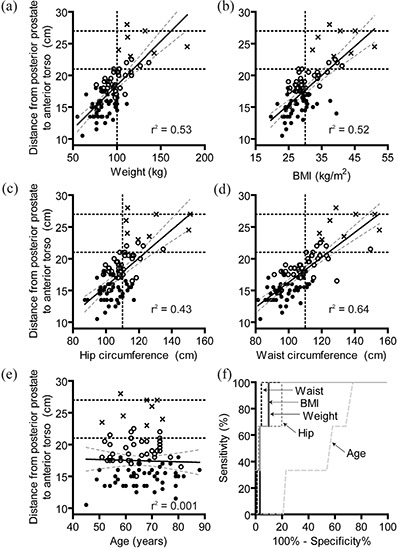
Scatter plot of weight (a), BMI (b), hip circumference (c), waist circumference (d), and age (e) vs. the distance from the posterior prostate to the anterior torso. Best fit line (solid) and 95% confidence intervals (dotted lines) in addition to linear regression goodness of fit parameter $\left(r^{2}\right)$ are shown for each plot. Based on manufacturer‐recommended surface measurements: those appropriate for localize and track (solid circles), localize only (open circles), or ineligible for Calypso (crosses) are indicated. The maximum depths that the Calypso system can provide tracking (21 cm) and localization (27 cm) are indicated by horizontal dashed lines. Vertical dashed lines represent selected cutoff value determined by ROC analysis. ROC curves (f) for age (light long dash), weight (solid dark), BMI (solid light), waist circumference (dashed dark), and hip circumference (dashed light).

Patient weight, BMI, waist circumference, and hip circumference were all well correlated with the distance from the posterior prostate to the anterior torso (Figs. 3(a–d) circles, open circles, and crosses, p < 0.0001) while age was not (Fig. 3(e), p = 0.79).

To determine a single cutoff value that could be used clinically, patients were categorized as either eligible (“localization and tracking” or “localization only”) or ineligible for Calypso based on the distance from the posterior prostate to the anterior torso. ROC curves were generated for weight, BMI, waist circumference, hip circumference, and age (Fig. 3(f)). Weight and waist circumference had the highest ROC area (0.95 and 0.97, respectively). Cutoff values were somewhat arbitrarily chosen based on reasonable sensitivity/specificity and easily measured values. The sensitivity/specificity of these metrics at selected cutoff values is shown in Table 2. Sensitivity and specificity of CT‐based greater trochanter to anterior torso measurements are included for comparison. For all four characteristics, there are cutpoints with a 100% sensitivity and a reasonable (e.g. > 65%) specificity.

**Table 2:** Receiver operating curve characteristics for patient eligibility for Calypso system use based on weight, BMI, waist circumference and hip circumference. The sensitivity and specificity associated with selected cutoff values are shown.

	*Area Under ROC Curve (95% CI)*	*Selected Cutoff Value*	*Sensitivity at Cutoff*	*Specificity at Cutoff*
Weight	0.95 (0.89–1.00)	100 kg	100	77
BMI	0.95 (0.90–1.00)	$30\;\text{kg}/m^{2}$	100	67
Waist Circumference	0.97 (0.94–1.00)	110 cm	100	69
Hip Circumference	0.93 (0.82–1.00)	110 cm	100	68
Greater Trochanter	1.00 (0.99–1.01)	23 cm	100	95

## IV. DISCUSSION

Men with prostate cancer often have several management options available to them.^(7)^ Given the lack of randomized trials comparing available treatment modalities, men often choose the treatment that combines their personal “best” risk‐to‐benefit ratio.^(8)^ Improved radiation targeting and patient setup can minimize the side effects associated with RT^(9)^ and alter the risk‐to‐benefit ratio in some patients.^(10)^ The Calypso system of electromagnetic transponders can be used for both radiation setup localization and real‐time tracking of prostate movement.

A unique aspect of the Calypso system is the ability to track the location of the prostate during treatment at a rate of 10 Hz. This allows a therapist to interrupt treatment if the prostate moves outside of preset limits and may allow for the use of smaller clinical target volume (CTV) to planning target volume (PTV) expansions.^(^ ^1^ ^,^ ^2^ ^,^ ^5^ ^)^ Improved precision and accuracy of treatment can decrease the dose of radiation delivered to the rectum and may minimize side effects associated with prostate radiotherapy.^(^ ^11^ ^,^ ^12^ ^)^ Whether the use of Calypso improves the therapeutic ratio for prostate radiotherapy is not known. Our data suggests that real‐time tracking may not be feasible in nearly 50% of patients (Fig. 2(b)), if transponders are placed in the posterior aspect of the prostate. This is a significantly higher percentage of patients than the 15% of patients ineligible for tracking in the multi‐institutional Calypso use report of Kupelian and colleagues.^(2)^ This difference may be due to the selection of thin, eligible patients in their study or to implantation of the transponders more anteriorly within the prostate that allows for tracking in a greater proportion of patients.

We have shown that when a patient lies supine, the vertical separation between the greater trochanter and anterior torso (as measured by cross sectional imaging) correlates strongly with the separation between the posterior edge of the prostate and the anterior torso. This distance is a good estimate for the maximum distance that a seed implanted within the prostate tissue will be from the array. This relationship agrees with a previous report describing the relationship between skeletal landmarks, topographic landmarks, and the dimensions of the prostate.^(13)^ It should be noted that this study was not designed to assess whether the greater trochanter to anterior torso measurement has any relationship to whether a patient is actually able to be localized using the Calypso system. In preclinical testing, a separation between the transponder and array of 27 cm of saline resulted in submillimeter accuracy in localization, and an array to transponder distance of 23.4 cm was used to track a moving target with an error of less than 0.5 mm.^(3)^ Thus, the cutoff values recommended by the manufacturer (17 cm and 23 cm) may underestimate the useful range of the system, even when accounting for a several centimeter gap between the array and the patient.

Based on ROC curves and ease of ascertainment, our data would suggest that patients weighing less than 100 kg do not need additional measurements taken to confirm that they are candidates for Calypso seeds. In nearly all clinics, obtaining a patient's weight is a standard component of the initial consultation and thus requires no additional work. BMI, hip circumference, and waist circumference are similarly well related to prostate depth, but appear not to provide additional predictive value beyond weight. Greater trochanter to anterior torso measurements have good sensitivity and specificity in predicting for the feasibility of Calypso use and, as suggested by the manufacturer, should be used in patients weighing more than 100 kg to quickly screen patients for Calypso use. Although the prostate can move independent of the pelvic bones,^(14)^ bony anatomy is a somewhat valid predictor of prostate depth for the purposes of selecting patients for Calypso fiducial use. This measurement may, however, inappropriately label a patient ineligible for Calypso. Those deemed ineligible on the basis of the greater trochanter to anterior torso measurement may benefit from additional imaging (e.g. CT to better define the distance from the planned fiducial placement to the anterior torso).

One of the challenges in implementing new technologies in oncology is determining which patients will benefit from the “advances”. Capital investment in equipment that will be used frequently, improve efficiency of care, or improve patient outcome is easier to justify. In this report, the majority (81.9%) of prostate cancer patients treated would have been candidates for radiation setup localization using the Calypso system. While it is not clear whether this system will improve the clinical workflow relative to alternative methods of isocenter localization (e.g. mV or kV port films with radio‐opaque markers, CT on‐rails, cone‐beam CT, or ultrasound^(^ ^15^ ^–^ ^20^ ^)^), it has been estimated that daily patient setup using Calypso takes less than one minute.^(2)^ This modest increase in setup time will likely be offset by a reduction in time needed for X‐ray‐based imaging.

Calypso transponders are often implanted by urologists. Knowledge of whether a given patient is appropriate for Calypso use could improve the urologist's workflow within the setting of a busy clinic. We have shown that clinicians can rapidly make reasonable decisions regarding suitability for Calypso based on simple clinical parameters (e.g. height and weight). While clinicians could use a diagnostic CT to determine the prostate depth and the appropriateness of Calypso, many patients receiving prostate radiation have early‐stage disease and have not had a pelvic CT prior to their simulation. In fact, NCCN guidelines and American College of Radiology Appropriateness Criteria do not recommend pelvic imaging for patients with early‐stage disease (i.e. a probability of lymph node involvement<20%).^(^ ^21^ ^,^ ^22^ ^)^ Therefore, use of a diagnostic scan to assess for Calypso appropriateness would require an additional scan with the accompanying cost, radiation exposure, and inconvenience. It has also been suggested that physicians would simply implant the Calypso marker in most patients and use kV X‐ray localization for those patients whose transponders were too deep for electromagnetic localization. At a cost of approximately $1,200 per Calypso beacon implantation, this represents a large waste of medical resources when compared to the cost of sterile gold fiducial markers ($27, IZI Medical Products, Baltimore, MD, Cat #GF1003, www.izimed.com).

We acknowledge several limitations of our study. First, there may be a bias due to inclusion of only patients who received radiation treatments for prostate cancer. However, a recent report based on the Cancer of the Prostate Strategic Urologic Research Endeavor (CaPSURE) database suggests that nonsurgical treatment approaches, such as radiation, are more commonly used in obese men with prostate cancer.^(23)^ Thus, a larger fraction of all patients with prostate cancer may be candidates for Calypso. Indeed, the mean BMI in our group $\left(29.7\;\text{kg}/m^{2}\right)$ is relatively high compared to the national average of $27.8\;\text{kg}/m^{2}$.^(24)^ This is consistent with higher rates of obesity $\left(\text{BMI}>29.9\;\text{kg}/m^{2}\right)$ in North Carolina (24.2%) compared to the national average (23.2%).^(25)^ Second, due to the retrospective nature of our study, waist and hip circumference measurements were done in the supine position and were based on the CT images. It is not known how well these CT‐based measurements correlate with hip and waist circumference measured in a different position, obtained as part of a physical exam per the manufacturer's recommendation, or estimated on the basis of patient reported measures. Our method of obtaining these measurements introduces some uncertainty into our approach, but also ensures consistency in measurements. The retrospective nature of our study does not allow us to compare greater trochanter to anterior torso separation determined by physical exam with that measured on CT. We suspect that physical exam‐based measurements would be less consistent that those obtained by cross sectional imaging. Finally, the number of patients studied is modest. As we continue to gain clinical experience treating patients using Calypso localization technology, we plan to continue evaluation and refinement of the “rules” reported here. Additional studies involving larger numbers of patients may help to clarify these issues.

## V. CONCLUSIONS

Based on the manufacturer's recommended surface measurements, 52% of patients were predicted to be eligible for both localization and tracking with Calypso. An additional 39% were predicted to be eligible for localization only. The distance between the greater trochanter and the anterior torso is an excellent surrogate for prostate depth. However, the measurement of the distance between the greater trochanter and anterior torso will underestimate the utility of Calypso. Patients weighing>100kg were uniformly eligible for the Calypso system, and thus weight is a useful metric for screening for Calypso appropriateness. In patients weighing ≥100kg, surface measurements or imaging of the prostate can be used to assess for Calypso appropriateness.

## ACKNOWLEDGEMENTS

RJK has been designated a B. Leonard Holman Pathway Fellow by the American Board of Radiology and is supported by a 2007–08 Phillips Medical Systems/Radiological Society of North America Research Resident Grant and by a 2008 Resident/Fellows in Radiation Oncology Seed Grant from the American Society for Therapeutic Radiology and Oncology. LBM receives grant support from the National Institutes of Health, Lance Armstrong Foundation, and Department of Defense. UNC Radiation Oncology receives grant support from Siemens Medical Solutions
